# Caregiving Network Changes Before and After a Care Recipient’s Transition to Residential Care

**DOI:** 10.1093/geroni/igaf122.1119

**Published:** 2025-12-31

**Authors:** Natasha Nemmers, Lu Qin, HwaJung Choi, Wenhua Lai, Amanda Leggett

**Affiliations:** Wayne State University, Detroit, Michigan, United States; University of Michigan, Ann Arbor, Michigan, United States; University of Michigan, Ann Arbor, Michigan, United States; Wayne State University, Detroit, Michigan, United States; Wayne State University, Detroit, Michigan, United States

## Abstract

This study examines the changes in caregiver network characteristics before and after an older adult transitions from community living to residential care. Although this transition is often assumed to reduce family involvement, evidence suggests that family caregivers remain actively engaged, adapting their roles to address evolving care demands. Using the National Health and Aging Trends Study (2011-2019), our sample included 7,197 Medicare-eligible older adults living in the community at baseline, 3,360 of whom later transitioned to residential care. We employed survey-weighted linear and multinomial logistic regressions to assess changes in caregiver network size, relationship types, and care task allocation, adjusting for demographic and health-related factors. Caregiver network size and shared responsibilities for medical, self-care/mobility, and household tasks gradually increased in the years preceding residential care admission, peaking one year before the transition, followed by a slight decline. A modest increase reemerged a few years post-admission. In contrast, shared transportation tasks remained relatively stable pre- and post-admission. Key caregivers, particularly daughters and other immediate family members, remained highly involved even after admission. The use of paid caregivers gradually increased over time, while non-family caregivers (e.g., neighbors, friends) declined immediately following admission but became involved again years later. The increase in caregiving support likely mirrors declining health or reduced independence in managing daily tasks. Family caregivers remain central, complementing the growing role of paid professionals who often handle more complex tasks. These findings highlight the need for structured care coordination and policies that acknowledge family caregivers’ enduring contributions during this pivotal transition.

